# Glutamatergic and Serotonergic Modulation of Rat Medial and Lateral Orbitofrontal Cortex in Visual Serial Reversal Learning

**DOI:** 10.1037/pne0000221

**Published:** 2020-06-04

**Authors:** Mona E. Hervig, Louise Piilgaard, Tadej Božič, Johan Alsiö, Trevor W. Robbins

**Affiliations:** 1 Department of Psychology, University of Cambridge, and Department of Neuroscience, University of Copenhagen; 2 Department of Psychology, University of Cambridge, and Behavioral and Clinical Neuroscience Institute, University of Cambridge

**Keywords:** serotonin, glutamate, orbitofrontal cortex, prefrontal cortex, reversal learning

## Abstract

Adapting behavior to a dynamic environment requires both steadiness when the environment is stable and behavioral flexibility in response to changes. Much evidence suggests that cognitive flexibility, which can be operationalized in reversal learning tasks, is mediated by cortico-striatal circuitries, with the orbitofrontal cortex (OFC) playing a prominent role. The OFC is a functionally heterogeneous region, and we have previously reported differential roles of lateral (lOFC) and medial (mOFC) regions in a touchscreen serial visual reversal learning task for rats using pharmacological inactivation. Here, we investigated the effects of pharmacological overactivation of these regions using a glutamate transporter 1 (GLT-1) inhibitor, dihydrokainate (DHK), which increases extracellular glutamate by blocking its reuptake. We also tested the impact of antagonism of the serotonin 2A receptor (5-HT_2A_R), which modulates glutamate action, in the mOFC and lOFC on the same task. Overactivation induced by DHK produced dissociable effects in the mOFC and lOFC, with more prominent effects in the mOFC, specifically improving performance in the early, perseveration phase. Intra-lOFC DHK increased the number of omitted responses without affecting errors. In contrast, blocking the 5-HT_2A_R in the lOFC impaired reversal learning overall, while mOFC 5-HT_2A_R blockade had no effect. These results further support dissociable roles of the rodent mOFC and lOFC in deterministic visual reversal learning and indicate that modulating glutamate transmission through blocking the GLT-1 and the 5-HT_2A_R have different roles in these two structures.

## Public Significance Statement

This study further supports dissociable roles of specific orbitofrontal subregions, as well as glutamatergic and serotonergic transmission in these subregions, in cognitive flexibility. This knowledge will add to the understanding of specific neural mechanisms underlying inflexible behaviour across psychiatric disorders.

Cognitive flexibility, the ability to adapt behavior in response to a changing environment, is disrupted in several psychiatric and developmental disorders including obsessive-compulsive disorder (OCD), schizophrenia, and autism (Chamberlain et al., 2008; D’Cruz et al., 2013; Leeson et al., 2009; Waltz & Gold, 2007). In OCD patients, inflexible behavior is typically treated with selective serotonin reuptake inhibitors, though typically not with full remission and with a large subgroup of nonresponders (Robbins, Vaghi, & Banca, 2019). Thus, more recently, drugs modulating cortical glutamate neurotransmission have gained attention, appearing beneficial in improving cognitive flexibility in OCD patients (Marinova, Chuang, & Fineberg, 2017). However, the underlying neural mechanisms of glutamatergic and serotonergic modulation of flexible behavior are not yet understood and need to be further investigated.

Flexible responding can be assessed in reversal-learning paradigms in humans (Fellows & Farah, 2003; Murphy, Smith, Cowen, Robbins, & Sahakian, 2002), monkeys (Butter, 1964; Dias, Robbins, & Roberts, 1996; Groman et al., 2013), and rodents (Chudasama & Robbins, 2003; McAlonan & Brown, 2003). In reversal learning tasks, initially learned reward contingencies change, and the subject needs to update behavior accordingly. This requires the subject to suppress prepotent responses, explore alternative options, learn new contingencies, and choose the previously unrewarded (but now rewarded) option. If the subject fails to adapt behavior, increased perseverative responding can occur (Iversen & Mishkin, 1970)—that is, “exploitation” of an outdated rule irrespective of diminished reward.

Reversal learning is thought to be mediated by cortico-striatal networks, with the orbitofrontal cortex (OFC) playing a prominent role, as supported by studies in rodents (Izquierdo, Brigman, Radke, Rudebeck, & Holmes, 2017), monkeys (Chau et al., 2015; Clarke, Robbins, & Roberts, 2008; Dias et al., 1996), and humans (Fellows & Farah, 2003; Ghahremani, Monterosso, Jentsch, Bilder, & Poldrack, 2010; Hampshire & Owen, 2006; Hornak et al., 2004; O’Doherty, Kringelbach, Rolls, Hornak, & Andrews, 2001; Rahman, Sahakian, Hodges, Rogers, & Robbins, 1999), including in OCD patients (Remijnse et al., 2006). In rodents, mostly the lateral OFC (lOFC) has been implicated in reversal learning (Alsiö et al., 2015; Bohn, Giertler, & Hauber, 2003; Burke, Takahashi, Correll, Brown, & Schoenbaum, 2009; Graybeal et al., 2011; Kim & Ragozzino, 2005; McAlonan & Brown, 2003; Schoenbaum, Chiba, & Gallagher, 1999, 2000; Schoenbaum & Setlow, 2003; Takahashi et al., 2009), but a functional medial-lateral dissociation in reversal learning is emerging (Dalton, Wang, Phillips, & Floresco, 2016; Hervig et al., 2020; Mar, Walker, Theobald, Eagle, & Robbins, 2011). In Hervig et al., we found that pharmacological inactivation of the lOFC impaired the early phase of visual reversal learning performance, while mOFC inactivation improved it, possibly reflecting a functional lOFC-mOFC balance in controlling exploration-exploitation strategies. Dissociable functions of lOFC versus mOFC have also been found in humans (Cheng et al., 2016; Dias et al., 1996; Elliott, Dolan, & Frith, 2000; Hampshire & Owen, 2006; Kahnt, Chang, Park, Heinzle, & Haynes, 2012; Kringelbach & Rolls, 2004; Milad & Rauch, 2012; Noonan, Chau, Rushworth, & Fellows, 2017; O’Doherty et al., 2001; Zald et al., 2014), including OCD patients (Apergis-Schoute et al., 2017; Gillan et al., 2015), and other primates (Noonan et al., 2010; Walton, Behrens, Noonan, & Rushworth, 2011).

Such dissociable roles of certain OFC subregions may be modulated by glutamate, the principal excitatory transmitter that regulates neuronal plasticity, learning, and memory (Gasbarri & Pompili, 2014). Prefrontal glutamate levels predict reversal learning performance in the marmoset monkey (Lacreuse, Moore, LaClair, Payne, & King, 2018). In rodents, aberrant function of the glutamatergic N-methyl-D-aspartate receptor (NMDAR) has been associated with impaired reversal learning (Brigman et al., 2008; Dalton, Ma, Phillips, & Floresco, 2011; Kumar, Olley, Steckler, & Talpos, 2015; Lobellova et al., 2013; Marquardt, Saha, Mishina, Young, & Brigman, 2014) and set-shifting (Svoboda, Stankova, Entlerova, & Stuchlik, 2015). Reversal learning deficits are also produced by aberrant NMDAR function in the lOFC (Brigman et al., 2013; Marquardt et al., 2019; Thompson, Josey, Holmes, & Brigman, 2015). The astrocytic glutamate transporter 1 (GLT-1) is responsible for more than 80% of total glutamate removal at the synapse, and it plays a key role in regulating cortical glutamatergic homeostasis (Danbolt, Storm-Mathisen, & Kanner, 1992; Gibbs, Hutchinson, & Hertz, 2008; Nieoullon, 2009; Petr et al., 2015). Reduced GLT-1 function increases extracellular glutamate levels and synaptic glutamatergic transmission (Roberts-Wolfe & Kalivas, 2015). Little work has been done to investigate the effects of increased glutamate availability following reduced GLT-1 function in reversal learning, but excessive glutamate impairs visual discrimination learning in GLT-1-deficient mice (Karlsson et al., 2009), as well as the initial stages of visual (Granseth, Andersson, & Lindström, 2015) and spatial (Balschun et al., 2010) reversal learning in vesicular glutamate transporter (VGluT1)-deficient mice. Such impairments are supported by excessive glutamate induced by global dihydrokainate (DHK) treatment impairing spatial memory (Bechtholt-Gompf et al., 2010) and memory retrieval (Tian, Yu, Cen, & Xiao, 2019).

Serotonin stimulates glutamate release in the prefrontal cortex, evoking excitatory postsynaptic potentials—a modulation mediated by serotonin 2A receptors (5-HT_2A_Rs; Aghajanian & Marek, 1999) that are robustly expressed in pyramidal neurons in the prefrontal cortex, including the OFC (Amargós-Bosch et al., 2004; Santana & Artigas, 2017; Santana, Bortolozzi, Serrats, Mengod, & Artigas, 2004), and predominantly in glutamatergic cells (Amargós-Bosch et al., 2004; Santana et al., 2004). Growing evidence suggests that serotonin promotes reversal learning behavior in rodents (Brigman et al., 2010; Brown, Amodeo, Sweeney, & Ragozzino, 2012; Zhukovsky et al., 2017), while reduced serotonin is associated with impaired reversal learning (Bari et al., 2010; Izquierdo et al., 2012; Lapiz-Bluhm, Soto-Piña, Hensler, & Morilak, 2009; Masaki et al., 2006; Matias, Lottem, Dugué, & Mainen, 2017), an effect also found following serotonin depletion in the OFC in monkeys (Clarke, Dalley, Crofts, Robbins, & Roberts, 2004, Clarke et al., 2005, Clarke, Walker, Dalley, Robbins, & Roberts, 2007; Rygula et al., 2015). Thus, in general, a vast amount of work suggests that reduced cortical (OFC) serotonin signaling increases perseveration and impairs reversal learning, whereas increasing serotonin generally facilitates reversal learning (Clarke et al., 2004), but serotonin exerts its effect through a variety of different receptors exerting both inhibitory and excitatory transmission depending on receptor subtype and localization.

In particular, the excitatory 5-HT_2A_Rs primarily localized on pyramidal neurons (Amargós-Bosch et al., 2004; Santana et al., 2004) and inhibitory 5-HT_2C_Rs primarily localized on inhibitory parvalbumin neurons (Liu, Bubar, Lanfranco, Hillman, & Cunningham, 2007) seem to be involved in reversal learning as systemic 5-HT_2A_R blockade impairs reversal learning performance, while systemic blockade of 5-HT_2C_Rs improves performance (Boulougouris, Glennon, & Robbins, 2008). While local 5-HT_2C_R antagonism in the lOFC reproduces this improvement, probably through inhibition of parvalbumin neurons leading to increased excitatory lOFC activity, intra-lOFC 5-HT_2A_R blockade does not affect spatial reversal learning (Boulougouris & Robbins, 2010). However, 5-HT_2A_R blockade with M100907 in the lOFC does impair odor-based reversal learning (Furr, Lapiz-Bluhm, & Morilak, 2012), and high levels of perseveration in rats are associated with decreased levels of 5-HT_2A_R in the lOFC and mOFC (Barlow et al., 2015), consistent with decreased levels of OFC 5-HT_2A_R predicting clinical severity in OCD patients (Perani et al., 2008). In the visual serial reversal learning paradigm used in the present study, intra-lOFC blockade of 5-HT_2C_R improves performance in the early phase of reversal learning (Alsiö et al., 2015), but the role of 5-HT_2A_Rs in the OFC still remains to be investigated on this task.

In the present study, we compared the effects of modulating glutamatergic transmission by DHK treatment and 5-HT_2A_R blockade in the lOFC and mOFC on a deterministic visual serial reversal learning in rats that we have previously shown to be dissociably affected by lOFC and mOFC inactivation (Hervig et al., 2020). We hypothesized that DHK-induced activation of the lOFC and mOFC would produce effects opposite to those of inactivating the lOFC and mOFC (Hervig et al., 2020). We further hypothesized that blocking 5-HT_2A_Rs would produce dissociable effects in the mOFC and lOFC, with hypothetical early reversal learning impairments in the lOFC as blocking the inhibitory 5-HT_2C_R in the lOFC produces early reversal learning improvements (Alsiö et al., 2015).

## Method

### Animals

Subjects were male Lister hooded rats (*N* = 42; Charles River, United Kingdom; Supplementary Table S1) housed in groups of three or four during behavioral pretraining testing and single-housed following guide cannulas implantation to protect the implant. The rats were housed under a reverse 12-hr light/dark cycle with lights off at 7:00 a.m. All training and testing were performed during the dark phase. To ensure sufficient motivation for task performance, the animals were food restricted with ad libitum access to water and fed once daily at random times after testing. Their body weights were maintained at 85% of their free-feeding weight. All experiments were subject to regulation by the United Kingdom Home Office (PPL 70/7548) in accordance with the Animals (Scientific Procedures) Act 1986.

### Drugs

M100907, or R-(+)-α-(2,3-dimethoxyphenyl)-1-[2-(4-fluorophenylethyl)]-4-piperidinemethanol (Sigma Aldrich, #M3324), a highly selective 5-HT_2A_ receptor antagonist (Kehne et al., 1996), was dissolved in 0.01 M phosphate-buffered saline (PBS) and 0.1 M hydrochloride and pH adjusted with NaOH to pH 6. M100907 was administered at 0 (vehicle), 1 μg/side, or 3 μg/side. DHK (Tocris Bioscience, United Kingdom, #0111), a potent blocker of GLT-1 (Arriza et al., 1994; Muñoz et al., 1987), was dissolved in saline and administered at 0 (vehicle/saline) and 1 μg/side.

Drugs were aliquoted in the quantities required for each test day and frozen at −80°C. For the intracranial microinfusions, the drugs were administered at 0.5 μl/side 10 min prior to testing.

### Behavioral Training (Touchscreen Serial Visual Reversal Learning)

**Figure 1 fig1:**
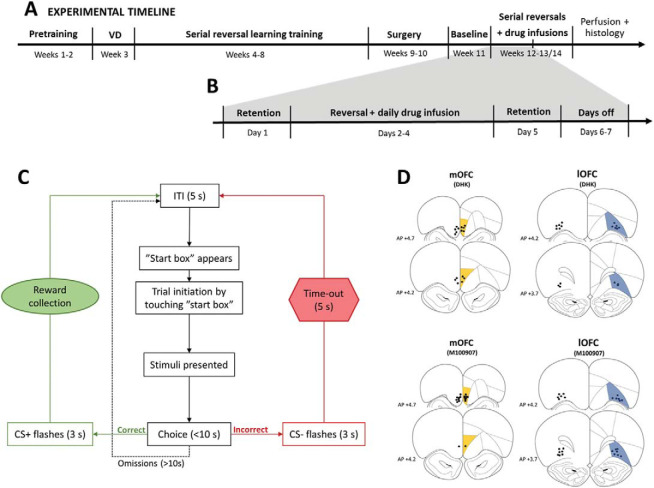
Experimental design—serial visual reversal learning. Panel A: Timeline of the touchscreen serial visual reversal learning experiment involving behavioral training, surgery, and behavioral testing with intracerebral infusions of DHK or M100907. Panel B: Timeline of one of the 2 (DHK) or 3 (M100907) weeks of reversal learning testing with drug or vehicle infusions. Panel C: Flowchart of possible trial sequences in the touchscreen visual discrimination and reversal learning task. Panel D: Schematic representation of brain sections showing the infusion sites in the mOFC (DHK, *N* = 8), lOFC (DHK, *N* = 8), mOFC (M100907, *N* = 9), and lOFC (M100907, *N* = 13) included in the reversal learning analyses. Infusion sites were characterized from brain sections prepared with cresyl violet. Coordinates are given as millimeter distance from bregma. CS = conditioned stimulus; ITI = intertribal interval or intertrial interval; VD = visual discrimination.

Behavioral training was performed as previously described in Hervig et al., 2020. For the experimental timeline and design, see Figure 1.[Fig-anchor fig1]

### Apparatus

We trained and tested the animals on a touchscreen serial visual reversal learning task using 16 operant chambers (Med Associates, Georgia, VT) placed in sound- and light-attenuating wooden cabinets equipped with a fan for ventilation and masking of external noise. The chambers measured 30 cm × 39 cm × 29 cm and consisted of a clear Perspex ceiling, front door, and back panel and metal paneling on the sides of the chamber. A metal grid with a removable metal tray below made up the floor of the chamber. A central food magazine coupled to an external pellet dispenser was located on one side of the chamber. It was equipped with light and infrared beam sensors to detect magazine entry, allowing delivery of one 45-mg sucrose pellet (TestDiet 5TUL; Sandown Scientific, Middlesex, United Kingdom) upon correct responses. A house light (∼3 W) was located near the ceiling directly above the magazine. A touch-sensitive screen (29 × 32 cm) presenting visual stimuli was located on the opposite side to that of the magazine. Task schedules were developed and implemented by A. C. Mar (Mar et al., 2013) using Visual Basic 2010 and have been published previously (Alsiö et al., 2015; Hervig et al., 2020).

### Pretraining: Touchscreen Serial Visual Reversal Learning

A five-stage pretraining phase began after the rats were food restricted, involving Pavlovian and instrumental conditioning prior to visual discrimination and serial reversal learning, and lasted until a stable baseline was reached. In Stages 1 to 3, rats were trained to respond to a single white box at the bottom center of the touchscreen for sucrose reward pellets during 60-min daily sessions until criterion of receiving the maximum 100 pellets in one session. The box decreased in size across the three stages until a final size of 3 × 4 cm (“start box”) in Stage 3. In pretraining Stages 4 and 5, two additional stimuli were introduced (horizontal and vertical bars). The first was at the bottom of the screen to ease touch (Stage 4); then, the stimulus was raised 5 cm to the final location on the screen to avoid accidental touches (Stage 5). At this point, touching the white start box was no longer reinforced but instead led to the presentation of one of these novel stimuli to the left or right (pseudorandomized location). Responding to the presented stimulus was reinforced with a sugar pellet, whereas responding to the blank side was signaled as incorrect by the illumination of the house light for a 5-s time-out period. Eighty percent or more correct touches on one stimulus in a session led to training sessions with the other stimulus. When criterion of > 80% correct touches was reached also on this stimulus, the rat moved on from Stage 4 to Stage 5, and after ≥ 80% correct touches were reached on both stimuli on Stage 5, visual discrimination training ensued.

### Visual Discrimination Training

In visual discrimination, the rats were presented with both stimuli simultaneously, of which one was reinforced. For session initiation, the rats would collect a free reward delivery, which led to presentation of the start box. The rat initiated a trial by responding to the start box, which initiated a simultaneous presentation of the stimuli pair. Responding to the correct stimulus (conditional stimulus; [CS]+) was reinforced with a sugar pellet, while responding to the incorrect nonreinforced stimulus (CS−) triggered a house-light-signaled 5-s time-out period. Failure to make a choice of either stimulus within 10 s of trial initiation was recorded as an omission. A 5-s intertrial-interval period preceded the next trial. To prevent the rats from developing a side bias, the stimuli were presented on the screen (left or right side) in a pseudorandom order (maximum three consecutive trials to the same side). The daily session ended after either 60 min, 150 rewards, or 250 trials, whichever was the first to occur. The rats reached criterion by 24 correct out of a running window of 30 trials. Prior to serial reversal learning training, a retention session with the same reward contingencies was given, as well as on the day following attainment of the learning criterion, to ensure that the rat had acquired the discrimination.

### Serial Visual Reversal Learning Training

Following the retention session during visual discrimination, the contingencies reversed so the rats then had to respond to the previous nonrewarded CS− stimulus (now CS+) for reinforcement until they reached the reversal learning criterion (24/30). A retention session both preceded and followed a reversal block. A stable serial reversal performance was achieved once the rat reached criterion within three consecutive daily sessions, with more than 200 trials completed on the first reversal day. The rats underwent surgery after they acquired a stable reversal learning performance.

### Stereotaxic Surgery

Rats were initially anesthetized with 5% isoflurane gas, which for the duration of the aseptic surgical procedure was reduced and maintained between 1% and 3%. We secured the rats in a stereotaxic frame (KOPF, Tujunga, CA) with atraumatic ear bars, set the tooth bar to −3.3 mm, and adjusted for flat skull position. Bilateral guide cannulas (22-GA; PlasticsOne, Roanoke, VA) were implanted in the lOFC (anteroposterior [AP] +3.5, mediolateral [ML] ±2.5, dorsoventral [DV] −1.7) and the mOFC (AP +4.0, ML ±0.6, DV −1.4) and secured with four screws and dental cement. Removable obdurators were inserted into the guide cannulas to prevent occlusion and protected with a dust cap. We obtained the surgical coordinates by using a stereotaxic atlas and made adjustments according to pilot surgeries. AP and ML coordinates were referenced to bregma, and DV was referenced to dura.

### Intracerebral Microinfusions and Reversal Learning Testing

Following the surgery recovery week, the rats were rebaselined on the serial reversal learning task to ensure a continued stable performance after the surgery. Following the baseline reversal week, which also included microinfusion habituation with sham infusions, we started the bilateral drug infusions of either M100907 or DHK across reversals according to a within-subject, crossover/Latin square design. The procedure was as follows: Prior to testing, the rats were gently restrained, and injectors (PlasticsOne; 28-GA) extending 2 mm below the guide were inserted into the guide cannulas. The injectors were left in place for 1 min before and after infusion, and drug was infused in a volume of 0.5 μl over 2 min. The rats were allowed to move freely around in the experimenter’s lap during infusion. Ten minutes after drug infusion, the rats were tested on the reversal task. Infusions were administered each day of reversal—that is, from the session when contingencies first shifted to the day criterion was reached, followed by a retention session with no infusion. Thus, an animal that reached criterion on the third day received three infusions across 3 consecutive days. On the day before the next reversal, another retention session was given in which the rats received saline infusion to ensure habituation to the infusion procedure as the rats typically had 2 days without testing between these retention sessions. Thus, a complete reversal with retention sessions and break took 7 days, during which the rats typically received three drug infusions.

### Histology

To confirm cannulas and injector-tip placements, we performed cresyl violet staining. Briefly, after the experiments, the rats were given a lethal dose of sodium pentobarbitone (Euthatal) and transcardially perfused with 0.01 M PBS followed by 4% (vol/vol) paraformaldehyde solution. The brains were removed, postfixed in 4% paraformaldehyde for 24 hr at room temperature, and dehydrated and preserved in 30% (wt/vol) sucrose in 0.01 M PBS for at least 2 days until sectioning. For sectioning, the brains were fast-frozen, embedded in optimal cutting temperature compound (O.C.T, VWR Chemicals, #361603E), and sectioned into 60-μm coronal sections using a cryostat (Leica, CM3050 S). The sections were stored in cryoprotectant at −20°C until cresyl violet staining.

### Experimental Design and Statistical Analyses

Only animals with intact cannulas during the course of the experiments and with correct regional placement of injector tips (see Figure 1) were included in the analyses (Supplementary Table S1). All experiments employed a within-subject complete crossover/Latin square design with separate cohorts for each region and drug. Data across days within one reversal were collapsed, and trial outcomes were coded as perseverative, random, or late learning depending on performance over bins of 30 trials in a rolling window, as described in detail and illustrated previously (Hervig et al., 2020), following binomial distribution probabilities (Jones & Mishkin, 1972). Postcriterion data (>24 correct) were excluded from analysis.

Behavioral data were subjected to analysis of variance (ANOVA) using a general linear model with significance at α = .05. Data were initially tested for normality with the Shapiro-Wilk test, and data that did not pass the Shapiro-Wilk test were appropriately transformed to obtain normal distribution before analysis (as described in further detail next). Outliers were tested by inspection of studentized residuals and would only be excluded from the analyses if the subject was consistently an outlier across all drug doses and behavioral phases; no animals were excluded. Homogeneity of variance was verified using Levene’s test; for repeated-measures analyses, Mauchly’s test of sphericity was applied to assure the sphericity assumption was not violated.

The dependent variables were trials, errors, reward collection and response latencies, omissions, as well as win-stay and lose-shift probabilities. Errors were square root transformed and analyzed to learning criterion and in each phase across regions. Lose-shift and win-stay probabilities were arcsine transformed and analyzed to criterion. Nonparametric testing was applied to analyze omissions in each phase and to criterion (Wilcoxon’s; note that omissions only occurred if the animals actively initiated a trial by touching the start box). Latencies to respond to the stimuli (after initiating a trial) and to collect earned reward pellets were analyzed to criterion.

To investigate whether treatment had an impact on the overall learning strategy, we analyzed the win-stay and lose-shift behavior as a proxy for learning from positive and negative feedback, respectively. We calculated the win-stay strategy as the probability of making a correct choice after a correct trial (*P*[stay|win]) and the lose-shift strategy as the probability of making a correct choice after an incorrect trial (*P*[shift|loss]; Clarke et al., 2008; Riceberg & Shapiro, 2012). Thus, *P*(shift|win) + *P*(stay|win) = 1 and *P*(shift|loss) + *P*(stay|loss) = 1.

The “criterion of learning” and “behavioral phase” data analyses across regions were performed with two-way mixed ANOVAs in a within-subject (Treatment) × between-subjects (Region) design for regional inactivation. Data were analyzed within each region using planned pairwise comparisons with Student’s *t* tests and repeated-measures one-way ANOVAs as appropriate.

All statistical analyses were performed using SPSS Version 25.0.0.1, and graphs were generated using GraphPad Prism 8 (San Diego, CA). Data are presented as mean ± standard error of mean. Significant effects will be *p* < .05, while *p* > .1 will be reported as noneffects. Effect sizes are indicated with partial eta squared (η_p_^2^; Cohen, 1988).

## Results

### Histological Assessment of Regional Infusion Sites

Of the 42 animals entering the reversal learning experiment, 38 rats were included in the analysis based on histological assessment of regional infusion sites. These comprised eight mOFC DHK, eight lOFC DHK, nine mOFC M100907, and 13 lOFC M100907 rats with correct regional injector placements (Figure 1D).

### Effects of DHK Infusion in the mOFC and lOFC on Reversal Learning

**Figure 2 fig2:**
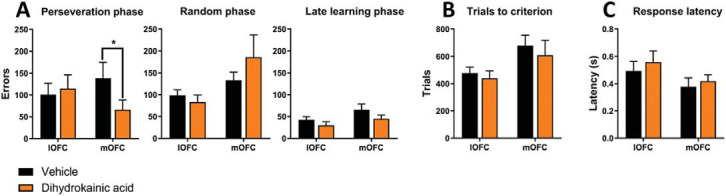
Effects of intra-OFC DHK infusions on performance in a deterministic touchscreen serial visual reversal learning task. Panel A: The effect of DHK microinfusion on errors within each reversal learning phase: perseveration, random, and late learning. Intra-mOFC DHK infusions improved reversal learning as reflected by a decrease in number of perseverative errors. Trials to criterion (Panel B) and response latencies (Panel C) were not affected by DHK infusions. Results are represented as mean ± standard error of mean. * *p* < .05.

Figure 2 shows that intra-OFC DHK produced dissociable effects on errors, with DHK in mOFC significantly reducing perseverative responses and intra-lOFC DHK not affecting errors, though significantly increasing omissions in the perseveration phase. For perseverative errors, ANOVA showed a significant DHK × Region interaction, *F*(1, 14) = 7.107, *p* = .018, η_p_^2^ = 0.34, and main effect of DHK, *F*(1, 14) = 4.69, *p* = .048, η_p_^2^ = 0.25, while there was no main effect of region, *F*(1, 14) = 0.054, *p* = .82, η_p_^2^ = 0.004 (Figure 2A). Planned pairwise comparisons within each region showed that intra-mOFC DHK significantly decreased the number of errors in the perseveration phase, *t*_7_ = 2.78, *p* = .027, η_p_^2^ = 0.524, while intra-lOFC DHK did not affect perseverative errors, *t*_7_ = −0.51, *p* = .628, η_p_^2^ = 0.35.[Fig-anchor fig2]

For the early learning (“random”) phase, ANOVA showed a main effect of region, *F*(1, 14) = 5.133, *p* = .040, η_p_^2^ = 0.27, but no DHK × Region interaction, *F*(1, 14) = 2.440, *p* = .141, η_p_^2^ = 0.148, and no main effect of DHK, *F*(1, 14) = 0.176, *p* = .681, η_p_^2^ = 0.012 (Figure 2A). For the late learning phase, ANOVA showed a significant main effect of DHK treatment, *F*(1, 14) = 4.869, *p* = .045, η_p_^2^ = 0.26, but no DHK × Region interaction, *F*(1, 14) = 0.001, *p* = .978, η_p_^2^ = 0.00006, or main effect of region, *F*(1, 14) = 2.317, *p* = .15, η_p_^2^ = 0.142. Planned pairwise comparisons within each region revealed no effects of DHK treatment on the late learning phase.

For trials to criterion, there was a trend toward a main effect of region, *F*(1, 14) = 4.53, *p* = .052, η_p_^2^ = 0.24, but no DHK × Region interaction, *F*(1, 14) = 0.58, *p* = .81, η_p_^2^ = 0.0041, or main effect of DHK, *F*(1, 14) = 0.74, *p* = .41, η_p_^2^ = 0.050 (Figure 2B). For omissions in the perseveration phase, a pairwise Wilcoxon signed-rank test showed that intra-lOFC DHK increased omissions, *p* = .043, while DHK in mOFC had no effect, *p* = .18 (Supplementary Figure S1). There were no effects in the random and late learning phases. We found no effects on response latencies (Figure 2C), collection latencies, or feedback sensitivity (Supplementary Figure S1).

In sum, DHK infused into the mOFC selectively reduced perseveration without affecting later learning phases. By contrast, DHK in the lOFC did not affect errors committed but increased omissions selectively in the perseveration phase.

### Effects of 5-HT_2A_R Blockade in the mOFC and lOFC on Reversal Learning

**Figure 3 fig3:**
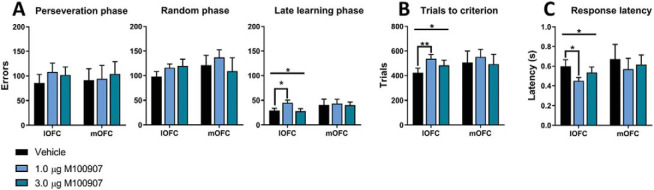
Effects of intra-OFC M100907 infusions on performance in a deterministic touchscreen serial visual reversal learning task. Panel A: The effect of M100907 microinfusions on errors within each reversal learning phase: perseveration, random, and late learning. Intra-lOFC M100907 infusions impaired late-stage reversal learning as reflected by an increased number of late learning errors. Intra-lOFC M100907 infusions increased trials to criterion (Panel B) and reduced response latencies (Panel C). Results are represented as mean ± standard error of mean. * *p* < .05. ** *p* < .01.

Intra-OFC 5-HT_2A_ blockade with M100907 produced dissociable effects on trials to criterion; intra-lOFC M100907 significantly increased overall trials required for learning, likely driven by increases across all phases, while intra-mOFC M100907 had no effect on trials (see Figure 3) but increased omissions overall (Supplementary Figure S2). For perseverative errors, ANOVA showed no M100907 × Region interaction, *F*(2, 40) = 0.24, *p* = .79, η_p_^2^ = 0.012, and no main effect of M100907, *F*(2, 40) = 0.34, *p* = .72, η_p_^2^ = 0.017, or region, *F*(1, 20) = 0.002, *p* = .97, η_p_^2^ = 0.000086, and there were no effects of M100907 when analyzing within each region (Figure 3A).[Fig-anchor fig3]

For the early learning (random) phase, ANOVA showed no M100907 × Region interaction, *F*(2, 40) = 1.41, *p* = .26, η_p_^2^ = 0.066, and no main effect of M100907, *F*(2, 40) = 1.54, *p* = .23, η_p_^2^ = 0.072, or region, *F*(1, 20) = 0.177, *p* = .68, η_p_^2^ = 0.009, and there were no effects of M100907 when analyzing within each region (Figure 3A). For the late learning phase, ANOVA across regions showed no M100907 × Region interaction, *F*(2, 40) = 0.71, *p* = .50, η_p_^2^ = 0.034, and no main effect of M100907, *F*(2, 40) = 1.66, *p* = .20, η_p_^2^ = 0.077, or region, *F*(2, 40) = 1.63, *p* = .22, η_p_^2^ = 0.075 (Figure 3A), although within each region, ANOVA showed a significant effect of M100907 in the lOFC, *F*(2, 24) = 4.48, *p* = .022, η_p_^2^ = 0.27, not the mOFC, *F*(1.9, 15.22) = 0.92, *p* = .90, η_p_^2^ = 0.011. Planned pairwise comparisons showed that 1 μg M100907 in lOFC significantly increased late learning errors, *t*_12_ = −2.65, *p* = .021, η_p_^2^ = 0.37, while 3 μg M100907 had no effect, *t*_12_ = −0.53, *p* = .61, η_p_^2^ = 0.023.

For trials to criterion, ANOVA across regions showed no significant effects. However, when analyzing each region, ANOVA showed a significant main effect of M100907 in lOFC, *F*(2, 24) = 3.96, *p* = .033, η_p_^2^ = 0.25, but not in mOFC, *F*(2, 24) = 0.24, *p* = .79, η_p_^2^ = 0.030. Planned pairwise comparisons within each region showed that 1 μg M100907 in lOFC significantly increased trials to criterion, *t*_12_ = −3.43, *p* = .005, η_p_^2^ = 0.49, while 1 μg M100907 in mOFC had no effect, *t*_8_ = −0.55, *p* = .60, η_p_^2^ = 0.037. There was no effect of 3 μg M100907 in lOFC, *t*_12_ = −1.48, *p* = .17, η_p_^2^ = 0.15, or mOFC, *t*_8_ = −0.14, *p* = .89, η_p_^2^ = 0.003.

For response latencies, ANOVA showed a trend toward a main effect of M100907, *F*(2, 40) = 2.58, *p* = .088, η_p_^2^ = 0.11, but no M100907 × Region interaction, *F*(2, 40) = 0.30, *p* = .75, η_p_^2^ = 0.015, or main effect of region, *F*(1, 20) = 0.23, *p* = .63, η_p_^2^ = 0.012 (see Figure 3). When analyzing each region, ANOVA showed a significant main effect of M100907 in lOFC, *F*(2, 24) = 3.58, *p* = .043, η_p_^2^ = 0.23, but not in mOFC, *F*(2, 16) = 0.49, *p* = .62, η_p_^2^ = 0.057. Planned pairwise comparisons showed that lOFC M100907 (1 μg) decreased response latencies, *t*_12_ = 2.92, *p* = .013, η_p_^2^ = 0.42, while there was no effect of the 3 μg M100907, *t*_12_ = 1.34, *p* = .21, η_p_^2^ = 0.13.

For omissions to criterion (Supplementary Figure S2), a pairwise Wilcoxon signed-rank test showed that 1 μg M100907 in mOFC significantly increased omissions, *p* = .031, while the lOFC M100907 did not affect omissions, *p* = .94. There was no effect of infusing 3 μg M100907 into the mOFC, *p* = .71, and lOFC, *p* = .22. There were no significant effects within the specific behavioral phases, although in the random phase, 1 μg M100907 in mOFC trended toward increasing omissions, *p* = .059. We found no effects on collection latencies or feedback sensitivity (Supplementary Figure S2).

In sum, 1 μg M100907 induced more effects than did 3 μg M100907, with these effects being found mainly in the lOFC. Intra-lOFC M100907 (1 μg) reduced reversal learning performance overall by increasing trials to criterion, probably driven by an increase in errors committed in the late learning phase. This reversal learning impairment was associated with faster response latencies. By contrast, 1 μg M100907 infused into the mOFC mainly increased omissions.

## Discussion

We observed dissociable effects of intra-OFC blockade of the GLT-1 following DHK (presumably resulting in increased extracellular glutamate) and of the 5-HT_2A_R with M100907 (presumably resulting in diminished 5-HT_2A_R-mediated glutamatergic transmission) on deterministic serial visual reversal learning. Intra-mOFC DHK reduced perseverative errors, while intra-lOFC DHK had no effect on errors committed. By contrast, intra-lOFC M100907 impaired overall reversal learning as reflected by increased trials required to reach the learning criterion—presumably driven by errors increasing cumulatively at each stage reaching significance during late learning. This impairment was also associated with faster response latencies. These results add to our previous finding of dissociable roles of the rodent mOFC and lOFC in visual reversal learning (Hervig et al., 2020), which has also been reported across other tasks such as probabilistic reversal learning (Dalton et al., 2016), delay discounting (Mar et al., 2011), and instrumental action (Gourley, Lee, Howell, Pittenger, & Taylor, 2010).

### Effects of Intra-OFC Blockade of GLT-1 on Serial Visual Reversal Learning

The present study shows that blockade of the astrocytic glutamate transporter GLT-1 with DHK in mOFC and lOFC affected reversal learning in a dissociable manner, though not in the direction that we expected. Based on our previous study (Hervig et al., 2020) showing that inactivating the lOFC impaired reversal learning, while inactivating the mOFC improved it, we expected to see somewhat opposite, and still dissociable, effects with DHK microinfusions. This is because DHK increases prefrontal extracellular glutamate levels (Pintor et al., 2004) and neuronal metabolic activity after local administration (at a comparable dose to the dose used in the present study) in the prefrontal cortex (PFC), while also decreasing related subcortical activity (Gasull-Camós et al., 2017). Thus, we predicted that microinfusion of DHK into lOFC would improve reversal learning, while it would impair reversal in the mOFC. Apparently paradoxically, intra-mOFC DHK improved reversal learning performance selectively in the early phase, as also occurred following inactivation of this structure. However, this improvement occurred in the absence of decreased collection latencies and enhanced negative feedback sensitivity produced by inactivation of the mOFC (Hervig et al., 2020). In contrast, intra-lOFC DHK had no effect on reversal learning performance.

Little work has been done on the role of the astrocytic GLT-1 in reversal learning, but dysfunctional astrocytes in the PFC impair reversal learning (Lima et al., 2014), and excessive glutamate impairs visual discrimination learning in GLT-1-deficient mice (Karlsson et al., 2009), as well as the initial stages of visual reversal learning (Granseth et al., 2015) and spatial reversal (Balschun et al., 2010) in VGluT1-deficient mice. Such impairments are supported by excessive glutamate induced by global DHK treatment impairing spatial memory (Bechtholt-Gompf et al., 2010) and memory retrieval (Tian et al., 2019). NMDAR antagonism, which results in excess glutamate and neuronal activation in the PFC (including the OFC; Hervig, Thomsen, Kalló, & Mikkelsen, 2016; Moghaddam, Adams, Verma, & Daly, 1997; Suzuki, Jodo, Takeuchi, Niwa, & Kayama, 2002), has also been reported to impair reversal learning in monkeys (Harder, Aboobaker, Hodgetts, & Ridley, 1998) and rodents (Abdul-Monim, Neill, & Reynolds, 2007; Dalton et al., 2011; Kumar et al., 2015; Li et al., 2016; Lobellova et al., 2013; Thonnard, Dreesen, Callaerts-Vegh, & D’Hooge, 2019; van der Meulen, Bilbija, Joosten, de Bruin, & Feenstra, 2003; Watson & Stanton, 2009)—an effect also seen in NMDA subunit-deficient mice (Brigman et al., 2008; Marquardt et al., 2014). Aberrant NMDAR function in the lOFC can also produce reversal learning deficits (Brigman et al., 2013; Marquardt et al., 2019; Thompson et al., 2015). However, there is some discrepancy as other studies with NMDAR antagonists either fail to show effects on reversal learning (Brigman, Ihne, Saksida, Bussey, & Holmes, 2009; Janhunen et al., 2015; Svoboda et al., 2015) or even show improvement in late-stage reversal learning (Dix, Gilmour, Potts, Smith, & Tricklebank, 2010; Fellini, Kumar, Gibbs, Steckler, & Talpos, 2014; McAllister, Mar, Theobald, Saksida, & Bussey, 2015). These contrasting findings may be due to methodological differences, for example, in terms of diverse pharmacological/genetic manipulations and the use of various reversal learning paradigms being dependent on different sensory modalities and, consequently, different brain structures. To understand these discrepancies, investigating effects of regional glutamate imbalance is necessary. In the present study, we show that excessive glutamate in the mOFC, and not the lOFC, improves visual serial reversal learning selectively in the early phase by reducing perseverative errors, without affecting response and reward collection latencies or feedback sensitivity. This observation supports a previous finding that prefrontal glutamate levels predict reversal learning performance in the marmoset, with enhanced glutamate availability being associated with better reversal learning performance (Lacreuse et al., 2018), suggesting that enhanced glutamate in certain PFC areas—for example, the mOFC—is beneficial for flexible responding.

We have previously suggested that the mOFC facilitates exploitative behavior (Hervig et al., 2020). DHK-induced excess glutamate in the mOFC likely disturbs the finely tuned glutamate homeostasis required for optimal neuronal functioning in learning and plasticity (Kalivas, 2009), in turn disrupting synchronized neuronal firing (Gray, 1994). This could hypothetically lead to inefficient cortico-striatal control over behavior and consequently enhanced exploration. This account may explain why intra-mOFC DHK to some degree mimics part of the effects from pharmacological mOFC inactivation on reversal learning observed previously (Hervig et al., 2020), while not fully reproducing those effects as the neural mechanisms are fundamentally different. As the mOFC, in contrast to lOFC, is the area most affected by DHK application in this study, it may be the region reflecting the brain circuitry responsible for the beneficial role of glutamate in reversal learning.

While it has been shown that optogenetic stimulation of lOFC-striatal projections suppresses compulsive grooming behavior (Burguière, Monteiro, Feng, & Graybiel, 2013), another study has shown that deep brain stimulation of the lOFC impairs spatial reversal learning, although not initial acquisition, in rats (Klanker, Post, Joosten, Feenstra, & Denys, 2013). Thus, the functional effect of lOFC activation on compulsive behavior is not straightforward, a conclusion further supported by the lack of effect of intra-lOFC DHK on visual reversal learning in the present study. As impaired reversal learning after lOFC inactivation or lesioning is well established across species, we expected to see some effect of “overactivating” the lOFC, but, at least in this paradigm, excessive glutamate in the lOFC does not seem to affect the lOFC’s control over dorsostriatal regions thought to be responsible for adapting behavior to altered response-reward contingencies in humans (Balleine & O’Doherty, 2010; Gillan et al., 2015; Morris et al., 2016), monkeys (Groman et al., 2013), and mice (Gremel & Costa, 2013). Alternatively, it is possible that overall glutamate excess does not affect lOFC neurons overall, but only subpopulations, due to the presence of functionally different individual neurons that exhibit different activational profiles depending on task after optogenetic stimulation (Jennings et al., 2019). Thus, variations in DHK infusion placements could in theory mask any specific effects mediated by individual lOFC neurons. This is further supported by studies showing that subpopulations of lOFC neurons exhibit task-dependent firing patterns during reversal learning (Gremel & Costa, 2013; Marquardt, Sigdel, & Brigman, 2017). At least, we can conclude that a hypothetical subpopulation effect in the lOFC is not transmitted to subcortical regions, such as the dorsolateral striatum, which is part of the neural circuitry mediating habitual learning (Gremel & Costa, 2013; Groman et al., 2013).

While intra-lOFC DHK did not affect primary measures of reversal learning performance, it did increase omissions specifically in the perseveration phase. Although this result should be interpreted with caution as it only encompasses few omissions in total, it does indicate some impairment in the early phase, possibly due to an attentional deficit resulting from hallucinatory-type actions (Jardri et al., 2016) or possibly due to some degree of anhedonia as shown for global and PFC DHK treatment in rats (Bechtholt-Gompf et al., 2010; John et al., 2012). Overall, our observations support a role for the GLT-1-mediated regulation of glutamate availability in the mOFC, not in the lOFC, in controlling reversal learning.

### Effects of Intra-OFC Blockade of the 5-HT_2A_R on Serial Visual Reversal Learning

We found that selective blockade of 5-HT_2A_Rs (by M100907) in the lOFC, not the mOFC, impaired reversal learning overall, as reflected by the increased number of trials required to reach learning criterion—an effect that presumably arose from increased errors committed cumulatively at each stage, reaching significance during late learning. This impairment was associated with faster response latencies, which could reflect overconfidence or impulsivity affecting decision-making. Blocking 5-HT_2A_Rs in the mOFC had no effect on reversal learning but increased omissions.

This finding is consistent with a role of orbitofrontal serotonin in reversal learning as previous studies have shown that serotonin and serotonin transporter levels/polymorphisms predict individual variation in reversal learning performance in rodents (Barlow et al., 2015; Lapiz-Bluhm et al., 2009; Stolyarova, O’Dell, Marshall, & Izquierdo, 2014) and monkeys (Groman et al., 2013; Vallender, Lynch, Novak, & Miller, 2009), that orbitofrontal serotonin depletion selectively impairs visual reversal learning in monkeys (Clarke et al., 2004, 2005, 2007; Rygula et al., 2015), associated with poor response suppression (Rygula et al., 2015), and that OFC serotonin is important for reinforcer devaluation (West, Forcelli, McCue, & Malkova, 2013). Our result is also consistent with previous systemic administration of M100907 impairing reversal learning performance on an operant two-choice spatial reversal learning task, whereas systemic blockade of 5-HT_2C_Rs had the opposite effect, improving performance (Boulougouris et al., 2008). While local 5-HT_2C_R antagonism in the lOFC reproduced this impairment, intra-lOFC 5-HT_2A_R blockade had no effect on spatial reversal learning (Boulougouris & Robbins, 2010). However, 5-HT_2A_R blockade with M100907 in the lOFC does impair odor-based reversal learning (Furr et al., 2012). Also, low reversal learning performance in rats is associated with decreased levels of 5-HT_2A_R, and serotonin, in the lOFC (Barlow et al., 2015), supporting our result that lOFC 5-HT_2A_R blockade impairs reversal learning performance.

In the visual serial reversal learning task used in the present study, intra-lOFC blockade of 5-HT_2C_R (primarily localized to inhibitory parvalbumin interneurons) improves performance in the early phase of reversal learning (Alsiö et al., 2015), which together with a reversal learning impairment after blockade of 5-HT_2A_R (primarily localized to glutamatergic pyramidal neurons) in the lOFC in the present study is consistent with 5-HT_2C_Rs controlling and 5-HT_2A_Rs facilitating reversal learning. However, it is important to note that as we did see an impairment, as expected, this impairment was not due to increased perseverative errors specifically, but rather an increase in errors committed across all reversal learning phases.

The discrepancy in effects of intra-lOFC M100907 administration on reversal learning is likely due to differences in reversal learning task design and sensory modalities involved. Boulougouris et al. used a spatial reversal learning task and only saw effects on the first, not the second or third, reversal the rats experienced, suggesting that novelty was also an important factor (Boulougouris et al., 2008; Boulougouris & Robbins, 2010). In the present study, we used a visual task, where the rats were trained in serial reversals to obtain stable reversal performance, allowing for within-subject analysis across reversals. Thus, our task is less dependent on circuitries involved in spatial cognition and excludes novelty as a possible factor.

It is important to note that only the lowest dose of 1 μg M100907 affected reversal learning significantly. This dose has been used in lOFC in a previous reversal learning study with no effects (Boulougouris & Robbins, 2010) and in the mPFC with effects on compulsivity (Mora et al., 2018). Both studies showed dissociable effects from intra-lOFC 5-HT_2C_R antagonist treatment—thus, this dose is presumably not targeting 5-HT_2c_R receptors. Also, in Furr et al. (2012), a dose comparable to the lowest dose in the present study impaired reversal learning when infused into the lOFC. However, no previous studies have used 3 μg M100907 in the OFC. Our results indicate that 3 μg M100907 had different effects from 1 μg, probably reflecting an inverted U-curve effect, which has also been reported for 5-HT_2A_R antagonists previously (Marek, Martin-Ruiz, Abo, & Artigas, 2005; Roth, 2011). Thus, our high dose may have induced receptor internalization, which is a known mechanism for the 5-HT_2A_R (Roth, 2011), supported by dose-response studies showing that systemic moderate doses of M100907 are more effective than low and high doses on a response inhibition task (Marek et al., 2005) and that intra-lOFC infusions with moderate M100907 doses induce the strongest detrimental effects on reversal learning compared with the low and high doses (Furr et al., 2012).

It is also worth speculating if the differential behavioral effects from the low and high doses of M100907 could be due to other factors. One factor is that the high M100907 dose could be targeting other receptors, in addition to the 5-HT_2A_R, to which M100907 has lower affinity, such as the 5-HT_2C_R. Since blocking the 5-HT_2C_R has opposite effects on reversal learning (Alsiö et al., 2015), this could in theory mask/counteract potential M100907 effects. However, this seems unlikely both because no opposite effects of low and high M100907 doses are observed in the early phase and because M100907 has subnanomolar affinity for 5-HT_2A_Rs, at least 100-fold lower affinity for 5-HT_2C_Rs, and negligible affinity for other receptors (Johnson, Siegel, & Carr, 1996; Kehne et al., 1996; Pehek, Nocjar, Roth, Byrd, & Mabrouk, 2006). It is also worth noting that, while the majority of prefrontal 5-HT_2A_Rs are postsynaptic, a small proportion are presynaptic (Cornea-Hébert, Riad, Wu, Singh, & Descarries, 1999; González-Maeso et al., 2007; Jakab & Goldman-Rakic, 1998; Miner, Backstrom, Sanders-Bush, & Sesack, 2003). The high dose of M100907 may, in theory, affect presynaptic, in addition to postsynaptic, receptors to a greater extent than the low dose and may thus modulate not only downstream neuronal excitation and long-term potentiation (Aznar & Klein, 2013) but also afferent neurotransmission (Barre et al., 2016). However, more investigations are needed to elucidate these potential underlying mechanisms. Moreover, although the findings were statistically significant with a high effect size within the lOFC, the lack of statistical significance and low to moderate effect sizes in the overall ANOVA indicate that this experiment eventually will require replication.

We hypothesize that the lOFC promotes exploration, and our present study suggests that 5-HT_2A_R in the lOFC may be responsible not so much for the initial switch from exploitation to exploration strategies that occurs at the time of reversal but for implementing the information acquired through exploration. Our study shows that the rats are able to initiate exploration (as perseverative errors are not statistically altered), but they commit increasingly more errors in the random (early learning) and late learning phases. Thus, the information acquired initially is not properly implemented in the existing task set encoded by the lOFC as more trials/errors to update, or create new, task sets were required following intra-lOFC M100907 treatment, consistent with the well-established role for the 5-HT_2A_R in learning and memory (Harvey, 2003; Zhang & Stackman, 2015). This impairment is not due to deficient feedback sensitivity as win-stay/lose-shift parameters were not affected by intra-lOFC M100907, but the observed speeding of response latency could reflect altered decision-making as a result of increased “guessing” before actually having made the correct decision. The OFC is involved in decision confidence and decision-making processes (Izquierdo, 2017; Kepecs & Mainen, 2012; Kepecs, Uchida, Zariwala, & Mainen, 2008) and required for optimal waiting based on decision confidence (Lak et al., 2014). Moreover, orbitofrontal serotonin depletion is associated with poor response suppression (Rygula et al., 2015), and prefrontal 5-HT_2A_Rs are thought to play a role in decision-making (Aznar & Klein, 2013).

That the reversal learning impairment is associated with faster responding may seem paradoxical, but this could be due to a potential role for 5-HT_2A_R and OFC in impulsivity. Although systemic 5-HT_2A_R antagonism decreases impulsive responding (Winstanley, Theobald, Dalley, Glennon, & Robbins, 2004), an effect presumably mediated through the nucleus accumbens (Robinson et al., 2008), an opposing role for 5-HT_2A_R in the lOFC is plausible.

### Concluding Summary

We found that increasing glutamate availability in the mOFC, not lOFC, improved early reversal learning, while blocking 5-HT_2A_Rs in the lOFC (presumably resulting in diminished glutamatergic transmission), not mOFC, lead to an overall impairment in visual reversal learning. These results further support dissociable roles of the rodent mOFC and lOFC in deterministic visual reversal learning and indicate that glutamate transmission and 5-HT_2A_R have different roles in these two structures.

## Supplementary Material

10.1037/pne0000221.supp
