# Supplementary material for: Glutamatergic and Serotonergic Modulation of Rat Medial and Lateral Orbitofrontal Cortex in Visual Serial Reversal Learning
Source: Psychol Neurosci. 2020 Jun 4;13(3):438–58. doi: 10.1037/pne0000221 (PMC7872199; doi:10.1037/pne0000221)
Supplement: Supplementary file 1 [file Supplementary_material_pne0000221.docx]

**Manuscript title: Glutamatergic and serotonergic modulation of rat medial and lateral orbitofrontal cortex in visual serial reversal learning**

Hervig ME, Piilgaard L, Božič T, Alsiö J and Robbins TW


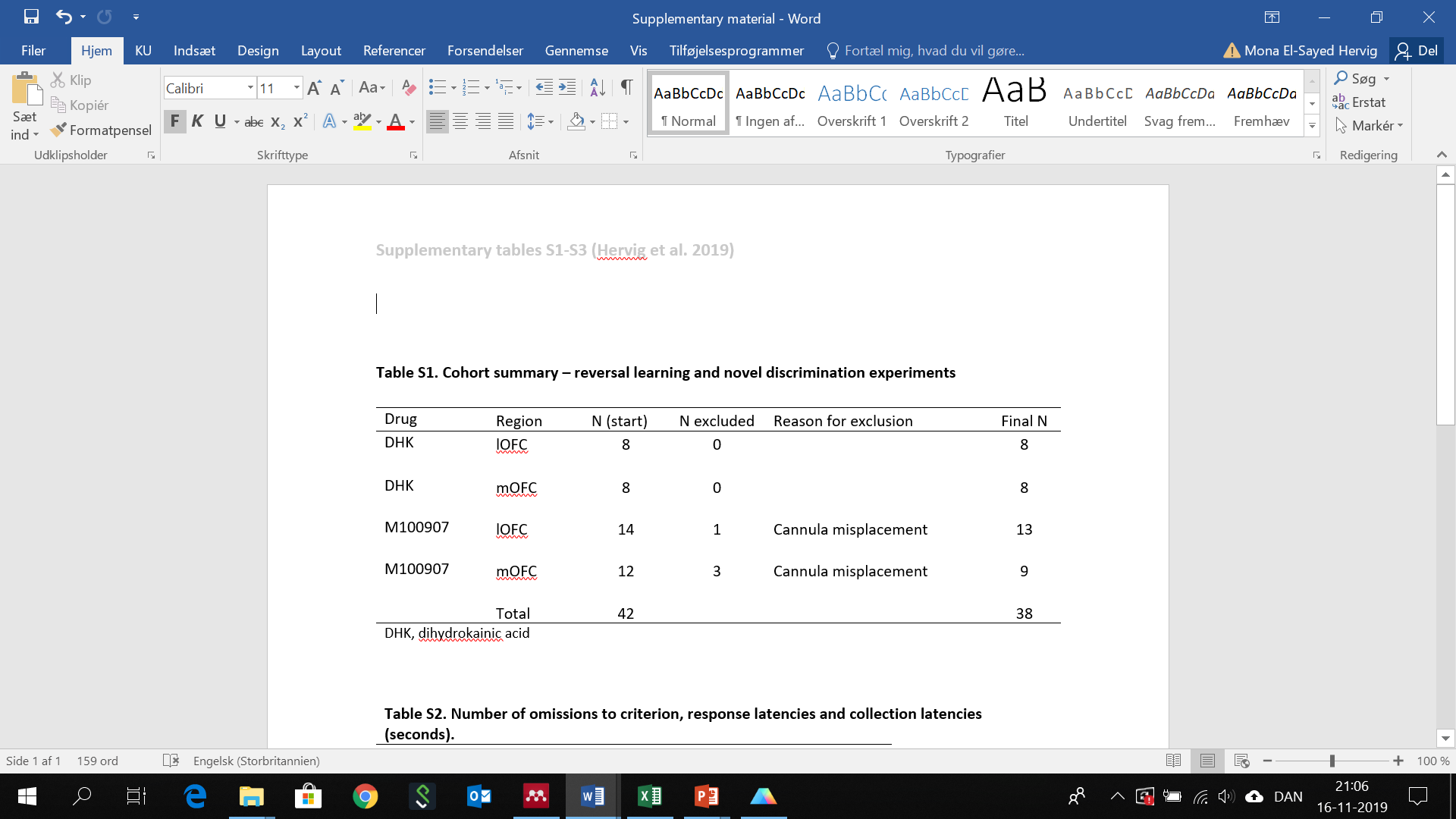


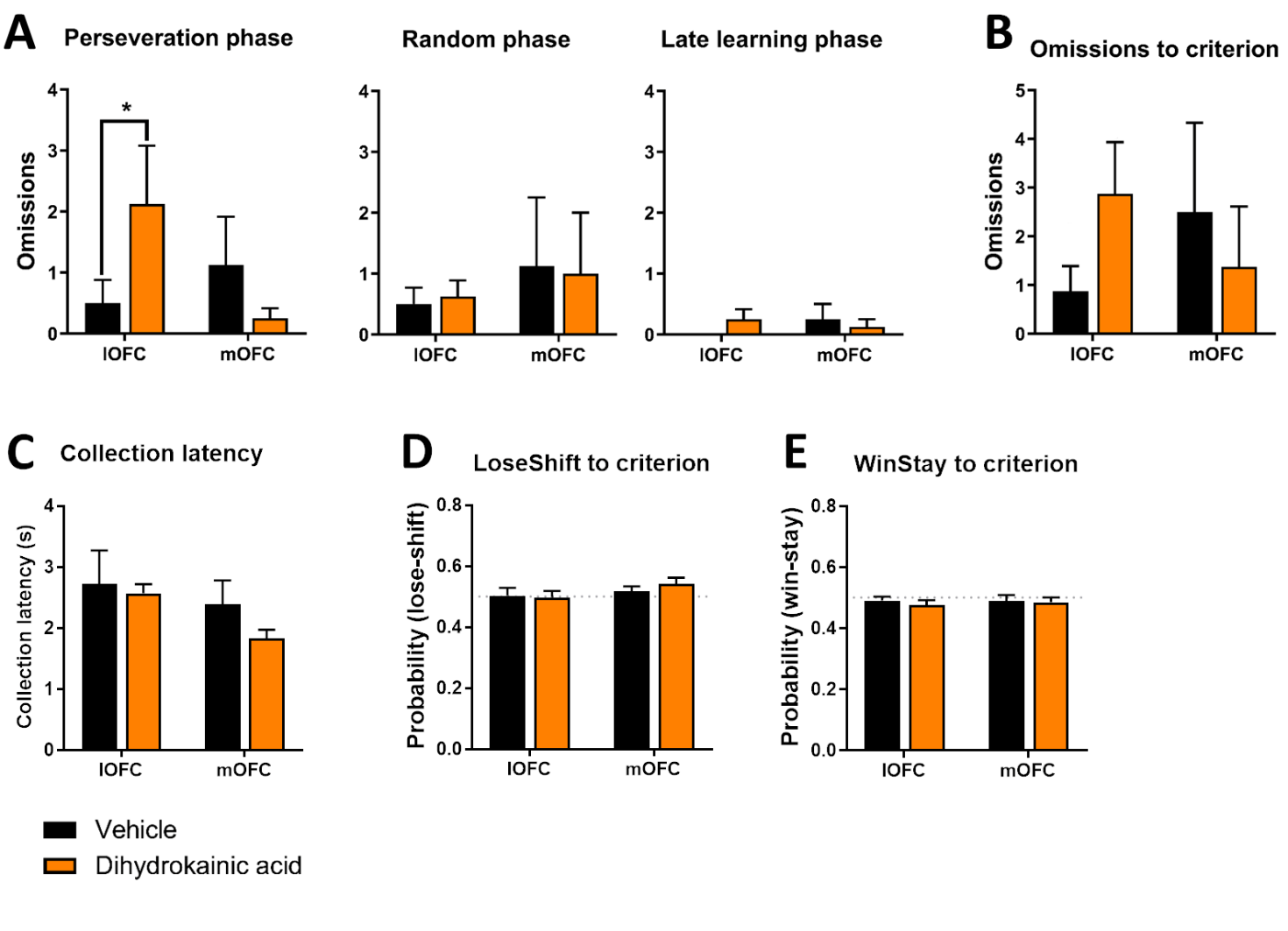


**Figure S1.** Effects of intra-OFC DHK infusions on omissions in a deterministic touchscreen serial visual reversal learning task. (A) The effect of DHK microinfusion on omissions within each reversal learning phase: perseveration, random and late learning. Intra-lOFC DHK infusions increased omissions in the perseveration phase. Omissions to criterion (B) and collection latencies (C) and feedback sensitivity (LoseShift (D) and WinStay (E)) were not affected by DHK infusions. Results are represented as mean ± SEM; **p* < 0.05.


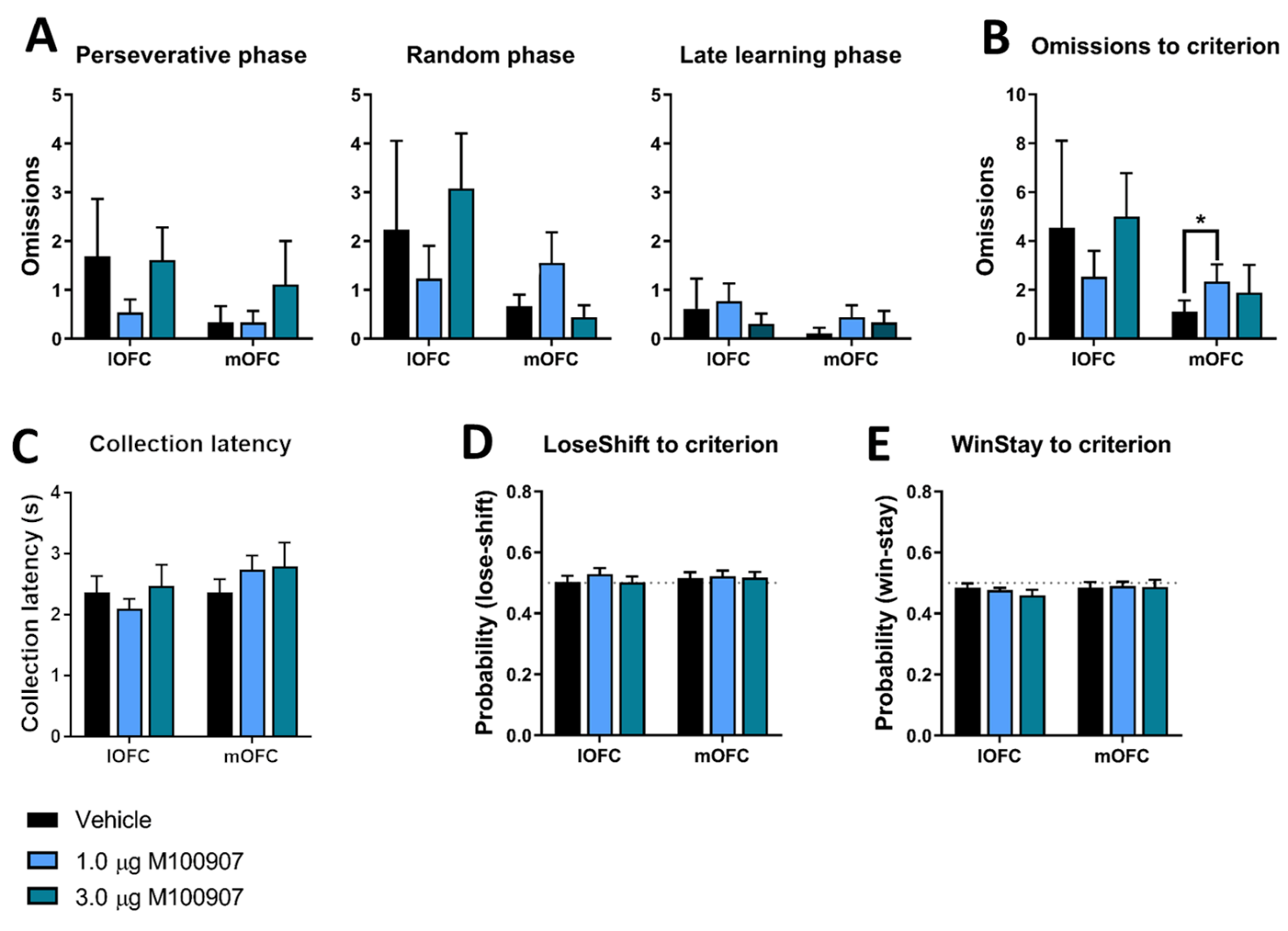


**Figure S2.** Effects of intra-OFC M100907 infusions on omissions in a deterministic touchscreen serial visual reversal learning task. (A) The effect of M100907 microinfusion on omissions within each reversal learning phase: perseveration, random and late learning. Intra-mOFC DHK infusions increased omissions to criterion (B). Collection latencies (C) and feedback sensitivity (LoseShift (D) and WinStay (E)) were not affected by M100907 infusions. Results are represented as mean ± SEM; **p* < 0.05.
